# Differential socioeconomic, psychosocial, and behavioral factors associated with psychological distress and uncontrolled blood pressure among women with and without HIV in the US

**DOI:** 10.3389/fmed.2025.1615684

**Published:** 2026-01-13

**Authors:** Jenni M. Wise, Emily B. Levitan, Elizabeth A. Jackson, Paul Muntner, Edgar T. Overton, Liang Shan, Jessica Blair, Andres Azuero, Jennifer H. McCarty, Maria L. Alcaide, David B. Hanna, Andrew Edmonds, Sheri D. Weiser, Seble G. Kassaye, Aruna Chandran, Gina Wingood, Deborah Konkle-Parker, Tracey E. Wilson, Kathleen M. Weber, Mirjam-Colette Kempf

**Affiliations:** 1 School of Nursing, University of Alabama at Birmingham, Birmingham, AL, United States; 2 Department of Epidemiology, School of Public Health, University of Alabama at Birmingham, Birmingham, AL, United States; 3 School of Medicine, University of Alabama at Birmingham, Birmingham, AL, United States; 4 Perisphere Real World Evidence, LLC, Austin, TX, United States; 5 ViiV Healthcare North America Medical Affairs, Durham, NC, United States; 6 Miller School of Medicine, University of Miami, Miami, FL, United States; 7 Department of Epidemiology and Population Health, Albert Einstein College of Medicine, Bronx, NY, United States; 8 Department of Epidemiology, Gillings School of Global Public Health, University of North Carolina at Chapel Hill, Chapel Hill, NC, United States; 9 School of Medicine, University of California, San Francisco, San Francisco, CA, United States; 10 Department of Medicine/Infectious Diseases, Georgetown University, Washington, DC, United States; 11 Bloomberg School of Public Health, Johns Hopkins University, Baltimore, MD, United States; 12 Mailman School of Public Health, Columbia University, New York, NY, United States; 13 Schools of Nursing, Medicine, and Population Health Sciences, University of Mississippi Medical Center, Jackson, MS, United States; 14 School of Public Health, SUNY Downstate Health Sciences University, Brooklyn, NY, United States; 15 Hektoen Institute of Medicine, Chicago, IL, United States

**Keywords:** HIV, MWCCS, blood pressure, hypertension, women

## Abstract

**Introduction:**

Women with HIV (WWH) have a higher risk of hypertension compared to women without HIV (WWoH). Exposure to adverse socioeconomic (e.g., area level deprivation) and psychosocial factors (e.g., stigma, inadequate social support) may contribute to inequities in hypertension through their influence on health behaviors (e.g., substance use, diet, physical activity) and psychophysiological (e.g., stress) responses.

**Methods:**

We examined the association between socioeconomic and psychosocial factors, psychological distress, and current uncontrolled blood pressure among WWH (*n*=998) and WWoH (*n*=353) enrolled in the Women’s Interagency HIV Study (WIHS) at a single visit between April and September 2019.

**Results:**

Socioeconomic and psychosocial factors were similar among WWH and WWoH. Among WWH and WWOH, 50.2% had current uncontrolled blood pressure, defined as a systolic blood pressure ≥130 mmHg or diastolic pressure ≥ 80 mmHg at the time of the study visit. Among WWH, socioeconomic, psychosocial, and behavioral factors explained 3% of the variance in blood pressure with self-reported health risk behaviors (*r*=0.15), and use of antihypertensive medication (*r*=0.09) had weak to moderate impact. Among WWoH, socioeconomic, psychosocial, and behavioral factors explained 10% of the variance in blood pressure, with self-reported health risk behaviors (*r*=0.19), use of antihypertensive medication (*r*=0.19), area-level social vulnerability (*r*=-0.17), and social support (*r*=0.16) demonstrating weak to moderate impacts.

**Discussion:**

Tailored interventions that address socioeconomic and psychosocial stressors at the individual and societal levels may improve outcomes and reduce disparities in uncontrolled blood pressure.

## Introduction

As HIV has become a chronic, manageable disease, people with HIV are living longer; however, they also have a higher prevalence of chronic diseases associated with aging, such as cardiovascular disease (1–3). Mortality rates from cardiovascular disease have more than doubled among people with HIV since 2000, and previous studies suggest that women with HIV (WWH) may be particularly vulnerable to hypertension and the associated morbidity and mortality from cardiovascular disease (4–6). A large cross-sectional analysis (*n =* 14,556,610) conducted using multiple medical record system platforms in the United States (US) demonstrated a high prevalence of hypertension among WWH (49%) compared to women without HIV (WWoH) (31%) (2). While it is well supported that the higher prevalence of hypertension among WWH can be partially attributed to HIV-specific factors (e.g., immune and inflammatory dysfunction and abnormal hormone production) (7, 8), less is known about the impact of socioeconomic and psychosocial factors on blood pressure and hypertension. Some studies suggest that in the context of HIV, increased exposure to adverse socioeconomic (e.g., poverty and food insecurity) and psychosocial (e.g., adverse childhood experiences, abuse, interpersonal violence, and intersectional stigma and discrimination) factors act as fundamental causes of inequities in hypertension and cardiovascular risk through their impact on health behaviors and stress (9–15). The purpose of this analysis was to examine the relationships between exposures (i.e., measured adverse socioeconomic and psychosocial factors, psychological distress, and health behaviors) and current blood pressure among WWH compared to sociodemographically similar WWoH. We hypothesized that WWH would experience exposure to adverse socioeconomic and psychosocial stressors, along with heightened symptoms of distress, and that these differences in exposures would contribute to the observed differences in blood pressure levels.

## Methods

The Women’s Interagency HIV Study (WIHS) was established in 1993 to study the overall effects of HIV among WWH in the US. Between 1993 and 2019, women were recruited across 10 geographically diverse sites in the US over 4 distinct enrollment periods. While enrollment criteria varied slightly across recruitment waves to keep pace with the evolving HIV epidemic in the US, WWH were eligible to participate if they were HIV-seropositive, assigned female at birth, and had not acquired HIV perinatally. WWoH were eligible if they were sociodemographically similar to WWH and reported high-risk behaviors or exposures associated with HIV transmission within the past 5 years (i.e., multiple sexually transmitted infections; unprotected sex with at least six individuals or any HIV-positive person; transactional sex for money, drugs, or shelter; or reported substance use, including crack, cocaine, heroin, methamphetamine, and other non-prescribed substances) (16). The inclusion of WWoH with similar sociodemographic and behavioral characteristics provides an opportunity to examine which outcomes are attributable to HIV infection versus other shared traits and experiences.

WIHS participants attended semiannual study visits to collect socioeconomic, psychosocial, behavioral, and clinical data. Nested within the framework of the overall WIHS study, the purpose of this study is to examine the association between socioeconomic, psychosocial, and behavioral factors and blood pressure levels among WWH and WWoH. The WIHS participants were included if they attended a study visit between April and September 2019, were 30–79 years of age at the time of the study visit, and had residential data available to link the cohort data with external data sources necessary to complete the analyses. The inclusion criterion of 30–79 years of age was selected based on the accelerated risk for developing cardiovascular disease among WWH compared to WWoH in the general population.

Blood pressure measurements were taken in a uniform manner across the study sites. Before taking the blood pressure measurements, the participants were asked to sit quietly with their feet flat on the floor for 5 min. An appropriate cuff size was selected based on the mid-arm circumference. An automated Dinamap Procare monitor was used to collect a series of three brachial blood pressure measurements at 1-min intervals. Measurements were collected from the right arm unless contraindicated, with the arm comfortably flexed and positioned at the heart level. The mean of three systolic and diastolic blood pressure values was calculated. A latent construct was created via exploratory factor analysis with systolic and diastolic pressure levels as manifest variables, and this construct is used as an outcome for bivariate and multivariable analyses. Current uncontrolled blood pressure was defined as systolic blood pressure of ≥130 mmHg or diastolic blood pressure of ≥ 80 mmHg at the time of study visit (17). Hypertension was defined as having a systolic blood pressure of ≥130 mmHg or a diastolic blood pressure of ≥ 80 mmHg, based on the average of two or more readings on two or more occasions (18). The definitions of current uncontrolled blood pressure and hypertension did not account for the reported use of antihypertensive medications. Instead, medication use was treated as a variable influencing blood pressure outcomes in our analyses.

**Figure 1:**
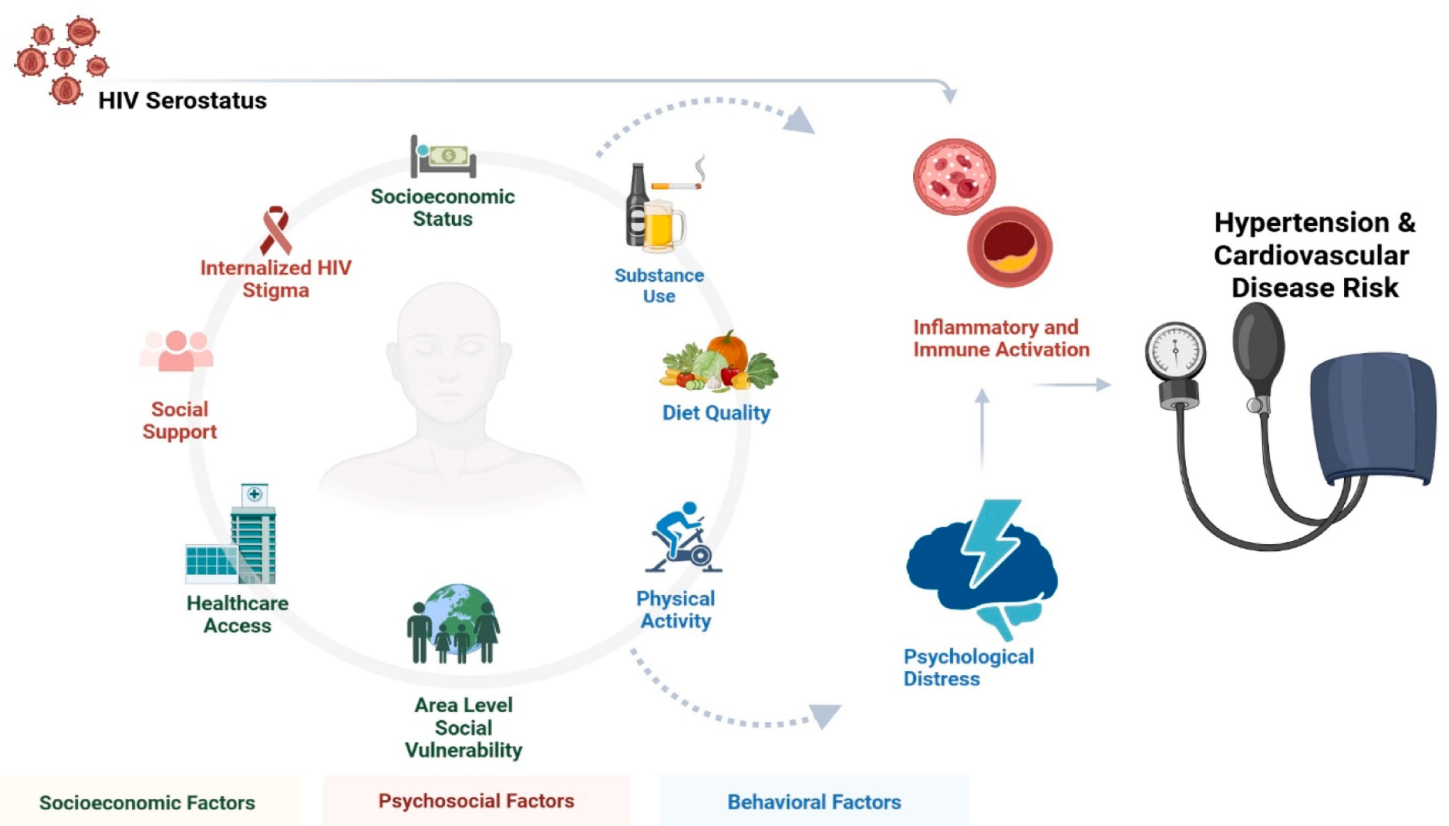
Socioecological model of stress and cardiovascular disease risk created in BioRender. Wise (54), https://BioRender.com/m74jtsw.

Predictors were informed by the Socioecological Model and the Stress Process Model, which outlined the hypothesized mechanisms and moderators linking adverse socioeconomic and psychosocial stress exposure to hypertension and cardiovascular disease risk (Figure 1).

### Socioeconomic factors

Self-reported residential addresses were geocoded and linked to census tract- and county-level variables to allow for the examination of area-level socioeconomic factors hypothesized to be associated with blood pressure. The Social Vulnerability Index (SVI) was used to characterize area-level social vulnerability based on social attributes (e.g., percentage of the population living below the poverty line, the unemployed, individuals without a high school diploma, those with disabilities, members of minoritized groups, individuals who speak English “less than well,” and residents of mobile homes, multi-unit housing, or crowded living conditions) associated with participants’ residential addresses at the census tract level using the 2018 dataset (19). The SVI scores range from 0 to 1 and reflect the relative ranking of each census tract compared to all US census tracts, with higher scores indicating greater vulnerability. Data from the Health Resources and Services Administration were used to assess access to primary care physicians (per 100,000 people) in relation to with participants’ residential addresses at the county level using the 2019 dataset.

Individual-level indicators of socioeconomic status were collected, including annual household income (≤$6,000, $6,001–12,000, $12,001–24,000, $24,001–36,000, $36,001–75,000, or >$75,000 per year), highest level of education attained (no school, ≤ 6th grade, ≤ 11th grade, high school, some college, completed 4-year college, and attended or completed graduate school), and participant’s enrollment in health insurance, ADAP, or Ryan White Programs (yes/no). To increase the interpretability of the data, we categorized annual household income into three groups (≤ $12,000, $12,001–24,000, and >$24,000 per year) and educational attainment into a single dichotomous option (≤ high school or > high school).

### Psychosocial factors

Psychosocial factors hypothesized to be associated with blood pressure included social support and internalized HIV stigma (20). The first 12 items of the Medical Outcomes Study (MOS) Social Support Survey were used to measure the perceived availability of emotional (e.g., someone to listen to me) and tangible (e.g., someone to help me if I am sick) social support among participants (21). A cumulative score was generated by averaging line-item scores (1–5), with higher scores indicating greater support. The Negative Self-Image subscale of the HIV Stigma Scale (22) was used to measure the extent to which participants with HIV internalize HIV stigma (e.g., feelings of self-shame or guilt) by averaging the line item scores (1–4). Higher scores indicate greater internalized stigma.

### Behavioral factors

Behavioral factors hypothesized to be associated with blood pressure included diet quality, weekly engagement in physical activity, reported use of cigarettes, alcohol use, and substance use (23–25). The 2000 National Health Interview Survey Dietary Screener was used to estimate the diet quality by approximating the intake of various food groups (e.g., fruits, vegetables, meats, and grains) and macronutrients (e.g., fat and fiber) among participants by day, week, month, and year. Diet quality scores (18–54) were created based on previously published estimates of nutrient composition by food group and the reported intake frequency of nutrient-rich foods (25, 26). The Physical Activity Questionnaire was used to measure the number of months participants engaged in weekly (≥2 h/week) mild (e.g., walking, bowling) or moderate activity (e.g., running, vigorous racquet sports) over the past 12 months, with one point assigned to each activity reported (27, 28). Cigarette smoking (yes/no) and consumption of alcoholic drinks per week (none, <7, 7–12, ≥12) were self-reported (29). Substance use (crack, cocaine, heroin, hallucinogens, methamphetamine, club drugs, or illicit use of methadone, amphetamines, narcotics, or tranquilizers) was self-reported (yes/no) since the last visit. Cannabis use (yes/no) was assessed separately to aid in the interpretation of the results.

### Psychological distress

Psychological factors hypothesized to be associated with blood pressure included depression, perceived stress, and post-traumatic stress symptom burden. The Centers for Epidemiological Studies Depression Scale (CES-D) was used to measure depressive symptoms, with scores ranging from 0 to 60, and scores ≥ 16 suggesting clinical depression (30). The Perceived Stress Scale was used to measure the degree to which everyday life was perceived as stressful, with scores ranging from 0 to 40, and scores >13 indicating moderate to high stress (31). The Post-traumatic Stress Disorder (PTSD) Checklist: The Civilian Scale was used to measure PTSD symptom burden, with scores ranging from 17 to 85, and scores >44 suggesting PTSD (32). A latent variable for psychological distress was created via exploratory factor analysis with symptoms of perceived stress, depression, and post-traumatic stress as manifest variables.

SAS 9.4 and R version 4.4.2. ® software packages were used to conduct the data analyses (33, 34). Descriptive statistics were calculated to characterize socioeconomic, psychosocial, and behavioral factors; psychological distress; and blood pressure outcomes (i.e., systolic and diastolic blood pressure, current blood pressure >130/80 mmHg, and reported use of antihypertensive medications). *t*-tests and chi-squared tests were used to examine the differences in HIV status.

### Dimension reduction

Principal component analyses (PCA) were conducted to reduce the dimension and increase the utility and interpretation of socioeconomic, psychosocial, and behavioral factors hypothesized to increase psychological distress and blood pressure. As certain predictors (e.g., HIV stigma and viral load suppression) are specific to WWH, separate analyses by HIV status were conducted to ensure a proper fit. Factors only applicable to WWH were recoded among WWoH to avoid missing data, with HIV stigma recoded to 0 and viral load to −10. PCA was conducted using all structural, social, and behavioral antecedents after recoding. To avoid the issue of imputed missing data resulting in different PCA solutions, a complete case analysis (*n* = 1,351) was conducted.

### Regression analyses

Simple linear regression analysis was conducted to examine the relationship between each factor, psychological distress, blood pressure, and uncontrolled blood pressure, separately and by HIV status. Multivariate regression was used to examine the impact of each factor on blood pressure. Assumptions were evaluated using diagnostic plots and deemed to be valid. No transformation was performed on the latent blood outcome. As predictors were informed by our conceptual model, which posits that socioeconomic, psychosocial, and behavioral factors influence blood pressure, we adjusted for the reported use of antihypertensives. As we hypothesized that psychological distress moderates the impact of socioeconomic, psychosocial, and behavioral factors on blood pressure, the interaction terms between the factors from PCA and psychological distress were entered into the model to examine the moderation effect of psychological distress on blood pressure levels.

## Results

A total of 1,351 women were included in the analyses, comprising 998 (74%) WWH and 353 (26%) WWoH (Table 1). The mean age of participants was 54.4 +/− 7.6 years. The majority (75%) of them identified as Black (non-Hispanic), with a higher percentage of WWH identifying as white (non-Hispanic) compared to WWoH (11% vs. 4%). The majority of women (73%) reported a household income of less than $24,000/year and lived in areas of greater social deprivation compared to the US national average. The average score on the Social Vulnerability Index was 0.73 +/− 0.25, indicating that 73% of the tracts in the US are less vulnerable than the average tract represented in this sample.

**Table 1:** Participant characteristics by HIV status.

Participant characteristics	Overall *N* = 1,351	Women without HIV *N* = 353	Women with HIV *N* = 998	*p*-value
	% (*N*) Mean ± SD	% (*N*) Mean ± SD	% (*N*) Mean ± SD	
Demographics				
Age, years	54.4 ± 7.6	54.2 ± 7.7	54.4 ± 7.6	0.694
Race, ethnicity				
Black, non-Hispanic	1,008 (74.5)	274 (77.6)	734 (73.5)	<0.0001
White, non-Hispanic	118 (8.7)	13 (3.7)	105 (10.5)	
Hispanic	168 (12.4)	43 (12.2)	125 (12.5)	
Other	57 (4.2)	23 (6.5)	34 (3.4)	
Socioeconomic factors				
Household income (USD/year)				
≤ $12,000	660 (48.9)	181 (51.3)	479 (48.0)	0.028
$12,001–24,000	326 (24.1)	67 (19.0)	259 (26.0)	
>$24,000	365 (27.0)	105 (29.7)	260 (26.1)	
High school graduate	897 (65.1)	236 (66.9)	643 (64.4)	0.411
Current employment	442 (34.9)	121 (34.3)	321 (32.2)	0.467
Health insurance^a^	1,306 (96.7)	315 (89.2)	991 (99.3)	<0.0001
Primary care physician access^b^	96.9 ± 35.0	101.1 ± 35.3	95.4 ± 34.8	0.017
Social vulnerability index [0–1]	0.73 ± 0.25	0.75 ± 0.25	0.73 ± 0.25	0.058
Psychosocial factors				
Emotional Social Support [1–5]	3.95 ± 1.05	3.95 ± 1.07	3.96 ± 1.05	0.906
Tangible Social Support [1–5]	3.9 ± 1.2	3.9 ± 1.2	3.9 ± 1.2	0.550
Internalized HIV Stigma [1–4]	–	–	1.8 ± 0.6	–
Health behaviors				
Diet Quality [18-54]^c^	37.9 ± 3.4	38.1 ± 3.3	37.9 ± 3.4	0.313
Mild Physical Activity^d^	0.8 ± 1.0	0.8 ± 1.0	0.8 ± 1.0	0.567
Moderate Physical Activity^d^	0.4 ± 0.8	0.4 ± 0.7	0.4 ± 0.8	0.986
Viral Suppression^e^			885 (88.7)	–
Cigarette Smoking	525 (38.9)	163 (46.2)	362 (36.3)	0.008
Substance Use^f^	112 (8.3)	48 (13.6)	64 (6.4)	<0.0001
Alcohol use				
Abstainer	701 (51.9)	155 (43.9)	546 (54.7)	0.001
1–7 drinks/week	525 (38.9)	152 (43.1)	373 (37.4)	
≥7–12 drinks/week	37 (2.7)	13 (3.7)	24 (2.4)	
>12 drinks/week	88 (6.5)	33 (9.3)	55 (5.5)	
Psychological distress				
CES-D Score [0–60]^g^	12.2 ± 11.5	12.3 ± 11.1	12.1 ± 11.6	0.337
PSS-10 Score [0–40]^h^	13.8 ± 8.2	15.0 ± 8.2	13.4 ± 8.2	0.003
PLC-C Score [17–85]^i^	33.3 ± 15.3	35.6 ± 15.8	32.4 ± 15.0	0.001
CES-D Score ≥16^j^	403 (29.8)	105 (29.7)	298 (29.9)	0.968
PSS-10 Score ≤ 13^k^	579 (42.9)	135 (38.2)	444 (44.5)	0.010
PCL-C Score > 44^l^	241 (19.8)	74 (22.7)	167 (18.7)	0.121
Blood pressure and medication use				
Systolic blood pressure, mmHg (mmHg)	129.8 ± 20.6	132.9 ± 21.8	128.7 ± 20.1	0.002
Diastolic blood pressure, mmHg (mmHg)	76.2 ± 11.1	77.3 ± 11.7	75.8 ± 10.8	0.037
Current uncontrolled blood pressure	678 (50.2)	193 (54.7)	485 (48.6)	0.050
Antihypertensive medication	772 (57.2)	205 (58.1)	567 (56.8)	0.574

^a^Health insurance, Ryan White, or ADAP enrollment. ^b^Physician access per 100,000 people. ^c^Lower scores indicate better quality diet. ^d^Activity reported ≥2 h per week over the last 12 months. ^e^HIV-RNA < 200 copies/ml. ^f^Reported use of crack, cocaine, heroin, hallucinogens, methamphetamine, club drugs, or the illicit use of methadone, amphetamines, narcotics, or tranquilizers since last visit. ^g^Center for Epidemiological Studies Depression Scale. ^h^Perceived Stress Scale. ^i^Post-traumatic Stress Disorder (PTSD) checklist for civilians. ^j^Suggesting clinical depression. ^k^Low perceived stress. ^l^Suggesting PTSD.

At the time of the study visit, 50% of all participants had current uncontrolled blood pressure (≥130/80 mmHg), and 19% had blood pressure readings ≥140/90 mmHg. Antihypertensive medication use was similar between WWH and WWoH (57% vs. 58%), both overall and among those with uncontrolled blood pressure (63% vs. 64%). Among those reporting antihypertensive medication use, WWH were more likely to have controlled blood pressure compared to WWoH (42% vs. 32%).

Psychosocial factors (i.e., emotional and tangible social support) appropriate for measurement among both WWH and WWoH were similar across groups. Internalized HIV stigma was only appropriate to measure among WWH. Differences existed in specific psychological distress factors according to HIV status. While no differences were present in depressive symptomology based on HIV status, roughly one-third (30%) of the women had CES-D scores ≥16, suggesting clinical depression. Differences existed in stress and post-traumatic symptom burden according to HIV status. WWH, compared to WWoH, were less likely to indicate “moderate or high” perceived stress (45% vs. 54%); however, they were more likely to report higher post-traumatic stress symptom burden (27% vs. 18%).

WWH were less likely than WWoH to smoke cigarettes (33% vs. 46%) and more likely to abstain from alcohol consumption (55% vs. 44%). A higher percentage of WWH reported substance use compared to WWoH (14% vs. 6%). Diet quality scores and physical activity were similar among participants, with the majority of participants reporting low levels of physical activity and diet quality scores reflective of those in the general population nationally (25).

### Dimension reduction

**Figure 2:**
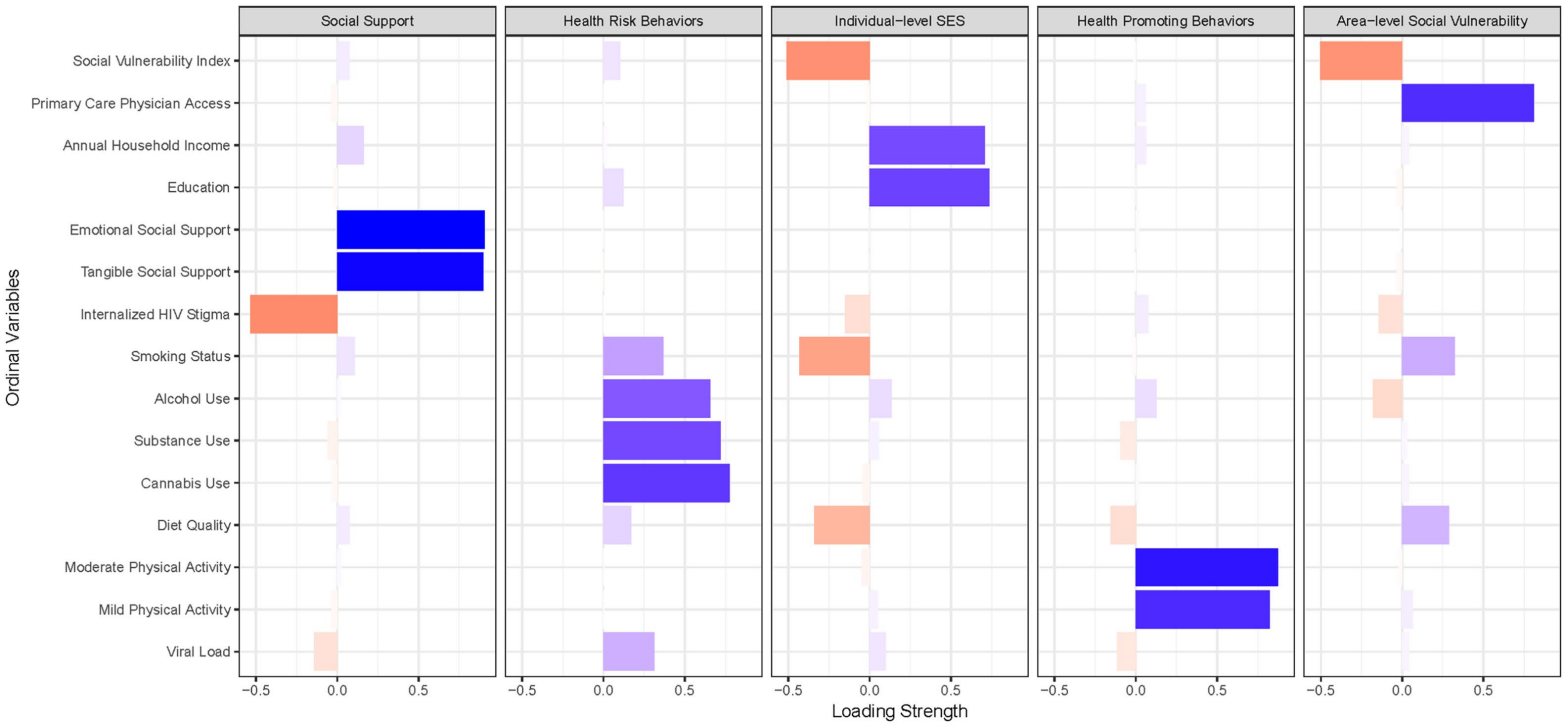
Principal component analysis factor loadings—women with HIV (*n =* 998).

**Figure 3:**
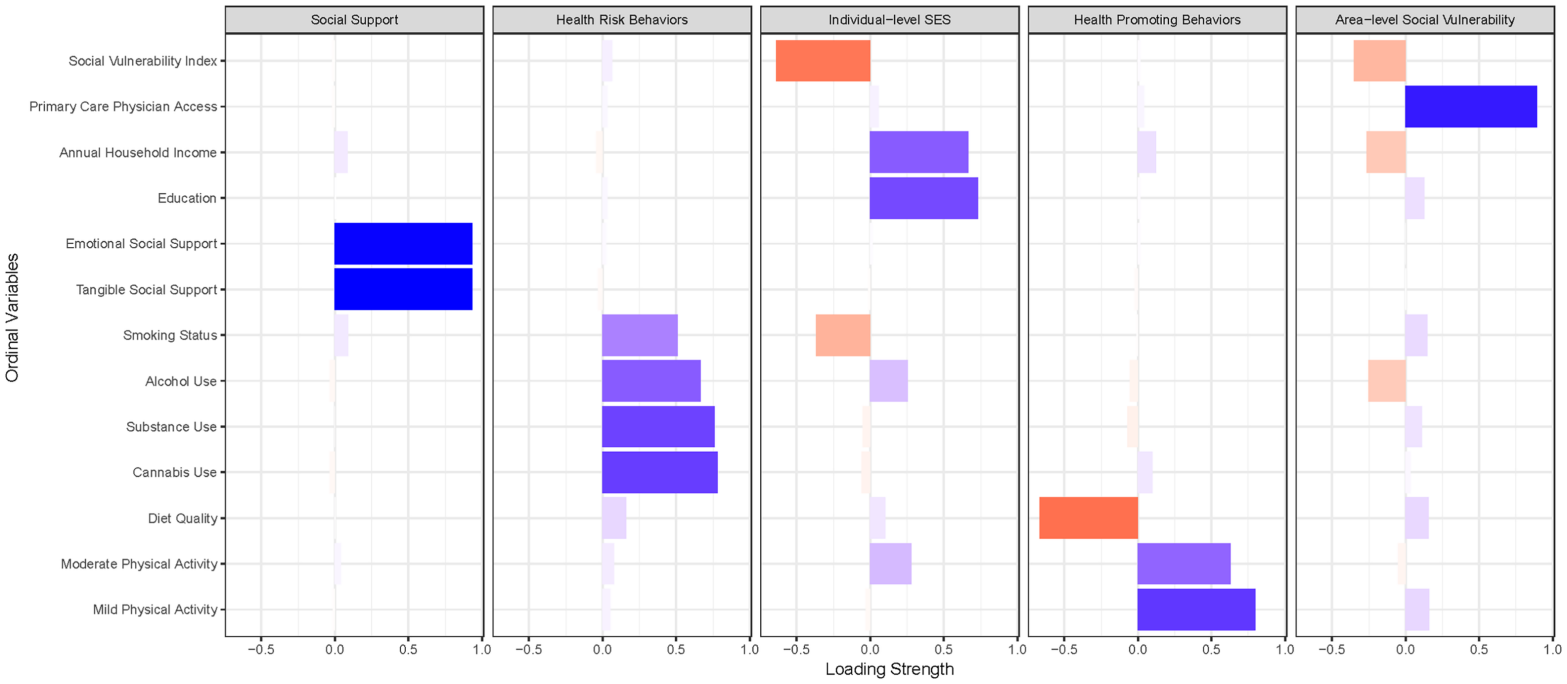
Principal component analysis factor loadings—women without HIV (*n =* 353).

Figures 2, 3 summarize the PCA factor loadings among all socioeconomic, psychosocial, and behavioral factors hypothesized to influence blood pressure and hypertension among WWH and WWoH. For both WWH and WWoH, five factors emerged: (1) individual-level socioeconomic status, (2) area-level vulnerability and physician care access, (3) social support, (4) health-promoting behaviors, and (5) health risk behaviors. The psychological distress construct generated had eigenvalue factor loadings of 0.88 for perceived everyday life stress, 0.86 for post-traumatic stress, and 0.58 for symptoms of depression. The total blood pressure construct generated had eigenvalue factor loadings of 0.71 for systolic blood pressure and 0.93 for diastolic blood pressure.

### Bivariate relationships between predictors, psychological distress, and blood pressure

Differences were observed based on HIV status (Table 2). For example, the relationship between post-traumatic stress symptoms and health risk behaviors was greater among WWH (*r =* −0.30) compared to WWoH (*r =* −0.17), whereas the relationship between area-level social vulnerability and perceived stress was greater among WWoH (*r =* 0.24) compared to WWH (*r =* 0.04).

**Table 2:** Correlations between socioeconomic, psychosocial and behavioral factors, psychological distress, and blood pressure.

Correlations between social- and behavioral factors, psychological distress, and blood pressure	Social support	Health risk behaviors^a^	Individual-level SES^b^	Health-promoting behaviors^c^	Area-level vulnerability	Depressive symptoms	Perceived stress	PTSD^d^ symptoms	Blood pressure	Uncontrolled blood pressure^e^
Women with HIV										
Social support	1.00	−0.12	−0.13	0.00	0.11	0.17	0.19	0.23	0.16	0.06
Health risk behaviors^a^	−0.12	1.00	0.02	0.01	−0.11	−0.53	−0.35	−0.30	−0.02	−0.03
Individual-level SES^b^	−0.13	0.02	1.00	0.24	0.06	−0.13	−0.18	−0.23	−0.06	−0.04
Health-promoting behaviors^c^	0.00	0.01	0.24	1.00	0.06	−0.04	−0.03	−0.04	0.01	−0.03
Area-level vulnerability	0.11	−0.11	0.06	0.06	1.00	0.05	0.04	0.06	0.04	0.00
Depressive symptoms	0.17	−0.53	−0.13	−0.04	0.05	1.00	0.51	0.50	0.02	0.01
Perceived stress	0.19	−0.35	−0.18	−0.03	0.04	0.51	1.00	0.75	0.07	0.01
PTSD symptoms	0.23	−0.30	−0.23	−0.04	0.06	0.50	0.75	1.00	0.09	0.04
Blood pressure	0.16	−0.02	−0.06	0.01	0.04	0.02	0.70	0.09	1.00	1.00
Uncontrolled blood pressure	0.06	−0.03	−0.04	−0.03	0.00	0.01	0.01	0.04	0.70	1.00
Women without HIV										
Social support	1.00	−0.10	−0.07	−0.06	0.15	0.13	0.13	0.15	0.13	0.02
Health risk behaviors^a^	−0.10	1.00	0.12	0.07	−0.07	−0.53	−0.35	−0.17	0.10	0.11
Individual-level SES^b^	−0.07	0.12	1.00	0.22	0.00	−0.19	−0.15	−0.18	−0.05	−0.06
Health-promoting behaviors^c^	−0.06	0.07	0.22	1.00	0.06	−0.14	−0.09	−0.10	−0.09	−0.08
Area-level vulnerability	0.15	−0.07	0.00	0.06	1.00	0.17	0.24	0.22	−0.12	−0.09
Depressive symptoms	0.13	−0.53	−0.19	−0.14	0.17	1.00	0.51	0.48	−0.04	−0.10
Perceived stress	0.13	−0.35	−0.15	−0.09	0.24	0.51	1.00	0.76	0.06	−0.01
PTSD symptoms	0.15	−0.17	−0.18	−0.10	0.22	0.48	0.76	1.00	0.12	0.02
Blood pressure	0.13	0.10	−0.05	−0.09	−0.12	−0.04	0.06	0.12	1.00	0.64
Uncontrolled blood PB^e^	0.02	0.11	−0.06	−0.08	−0.09	−0.10	−0.01	0.02	0.64	1.00

^a^Socioeconomic status; ^b^smoking, alcohol, and substance use; ^c^physical activity and diet quality; ^d^post-traumatic stress; mean blood pressure ≥130/80 mmHg at the time of the visit.

Differences were present in the relationship between predictor variables and blood pressure by HIV status. There was minimal evidence to support the relationship between predictor variables and blood pressure among WWH, with social support demonstrating a weak relationship (*r* = 0.16) with continuous blood pressure. Among WWoH, there were weak relationships between social support (*r* = 0.13), health risk behaviors (*r* = 0.10), area-level vulnerability (*r* = −0.12), and PTSD symptomology (*r* = 0.12) with continuous blood pressure, and weak relationships between health risk behaviors (*r* = 0.11) and depressive symptoms (*r* = 0.10) with current uncontrolled blood pressure.

### Multivariate relationships between predictors, psychological distress, and blood pressure

Among WWH, the factors hypothesized to be associated with hypertension explained 3% (adjusted R^2^ = 0.03, *p* < 0.001) of the variance in blood pressure, with health risk behaviors (*β* = 0.15, *p* < 0.001) and antihypertensive medications (β = 0.09, *p* < 0.01) having medium and weak effects, respectively (Table 3). There was no evidence of interactions between any factor and psychological distress.

**Table 3:** Multivariable relationships between structural, social, and behavioral factors and blood pressure.

Structural, social, and behavioral factors among women with HIV/Structural, social, and behavioral factors among women without HIV	Unstandardized Coefficients			Standardized Coefficients	*p*-value
	Beta^a^	95% CI		Beta^b^	
		(Lower)	(Upper)		
Women with HIV					
Social support	0.020	−0.046	0.086	0.022	0.549
Health risk behaviors	0.139	0.075	0.203	0.151	<0.001
Socioeconomic status	−0.031	−0.097	0.035	−0.034	0.337
Positive health-promoting behaviors	0.029	−0.035	0.093	0.031	0.432
Area-level vulnerability	0.024	−0.038	0.086	0.026	0.432
Antihypertensive medications	0.176	0.052	0.3	0.094	0.001
Psychological distress	0.057	−0.017	0.131	0.057	0.126
Women without HIV					
Social support	0.157	0.047	0.267	0.160	<0.001
Health risk behaviors	0.191	0.079	0.303	0.190	<0.001
Socioeconomic status	0.007	−0.105	0.119	0.007	0.896
Health-promoting behaviors	−0.082	−0.192	0.028	−0.083	0.136
Area-level vulnerability	−0.166	−0.276	−0.056	−0.166	0.001
Antihypertensive medication	0.395	0.177	0.613	0.195	<0.001
Psychological distress	0.162	0.036	0.288	0.153	0.001
Health-promoting behaviors*		0	0.24		
Psychological distress	0.120			0.108	0.010
Adjusted R^2^ = 0.10					

^a^Change in blood pressure based on a one-unit change in each independent variable. ^b^Relative strength (0–1) and direction of each independent variable of blood pressure.*This is the interactive effect between health promoting behaviors and psychological distress.

For WWoH, the factors included in the model explained 10% of the variance in blood pressure (adjusted R^2^ = 0.10, *p* < 0.001), with health risk behaviors (*β* = 0.19, *p* < 0.001), antihypertensive medication (*β* = 0.19, *p* < 0.001), socioeconomic status (*β* = −0.17, *p* < 0.01), and social support (*β* = 0.16, *p* < 0.01) having medium effects on the overall model. An interactive effect (*β* = 0.11, *p* < 0.05) was observed between health-promoting behaviors and psychological distress.

## Discussion

We hypothesized that, compared to WWoH, WWH would have increased exposure to adverse socioeconomic and psychosocial stressors and increased psychophysiological responses to stress and that these differences would be associated with uncontrolled blood pressure. Instead, we found that uncontrolled blood pressure was more prevalent among WWoH and was an unexpected explanation for the differences in outcomes based on our hypotheses. Among WWoH, adverse socioeconomic and psychosocial exposures, along with engagement in health risk behaviors, contributed substantially to variances in blood pressure, supporting the overall evidence that social determinants of health serve as fundamental causes of health disparities through their impacts on health behaviors. Although WWH had similar exposures to socioeconomic and psychosocial factors compared to WWoH, these factors appeared to play a less prominent role in affecting the variance in blood pressure in this group.

The higher prevalence of current uncontrolled blood pressure among WWoH in the WIHS cohort may reflect differences in healthcare access and engagement in care. Evidence supports that WWH in the general population has a higher prevalence of hypertension compared to WWoH (78% vs. 51%) (35). Involvement in the WIHS included semiannual study visits in which WWH had the opportunity for more intensive medical management and access to social services compared to the general population of WWH in the U.S. (16). While both WWH and WWoH, who are enrolled in research studies, likely receive more consistent and comprehensive care than their demographically similar counterparts in the general population who are not enrolled in structured research studies, WWoH do not qualify for HIV-specific programs (such as Ryan White or ADAP) that may additionally improve access to care. WWoH in the WIHS were much less likely to have access to affordable antihypertensive medications (through health insurance or enrollment in Ryan White or ADAP) compared to WWH (16). Nationally representative studies have previously demonstrated that uninsured individuals have less access to screening, preventative, and treatment services related to blood pressure management (36–39).

While the current results did not support the hypothesis that adverse exposures to socioeconomic and psychosocial factors influence differences in blood pressure through their impacts on psychological distress, several factors may explain these outcomes. Our results may underestimate the actual impacts of socioeconomic and psychosocial stressors on the lives of WWH, potentially due to survival bias or unmeasured resiliency characteristics (20, 40). Evidence suggests that when individuals are exposed to chronic stressors across their lifespan (e.g., adverse childhood events, interpersonal violence, economic instability, and intersectional stigma and discrimination), they may normalize or underreport psychological stress as an adaptive coping mechanism to improve quality of life (41). While women in our study, on average, lived in areas more vulnerable (characterized by low socioeconomic status, racial and ethnic minority status, and poor-quality housing) than 73% of all census tracts in the US, many (42.9%) reported relatively low everyday life stress. While the majority of women reported depression symptomology below the threshold suggesting clinical depression, prior research conducted within the WIHS has linked higher CES-D scores with overall mortality. In addition, these results highlight the differences between WWH enrolled in the WIHS and WWH in the general population, as evidence suggests that WWH experience psychological distress (e.g., high levels of chronic stress, depression, and PTSD) at rates 4–5 times higher than their seronegative counterparts (42, 43). The lower prevalence of psychological distress among WWH enrolled in the WIHS compared to WWH in the general population may reflect more consistent access to mental health care screening and referrals and increased access to peer support, counseling, and community resources through their involvement in the study (16, 20, 44).

While the overall prevalence of current uncontrolled blood pressure was relatively lower among WWH, 50% of all women included in our analyses had a current blood pressure level of ≥130/80 mmHg, making improved blood pressure control among both WWH and demographically similar WWoH a clinically and socially significant issue. Moreover, our results demonstrate that while the reported use of antihypertensive medications was similar according to HIV status, we found that antihypertensive medications were associated with higher blood pressure. Even with treatment, individuals with previously diagnosed hypertension often have higher blood pressure than those without underlying hypertension. Differences in these relationships between WWH and WWoH likely represent less adherence among WWoH in our sample. Our findings suggest a need for better blood pressure management among WWH and sociodemographically similar WWoH. Although standardized guidelines do not provide recommendations specific to people with HIV (7, 45), multiple studies have demonstrated that hypertension disproportionately increases the risk of cardiovascular disease morbidity and mortality among people with HIV (5, 7, 46, 47). The physiological impacts of HIV and treatments necessary for its management may compound the risk associated with exposure to adverse socioeconomic and psychosocial conditions, as well as psychological distress. Lifestyle modifications (e.g., stress, anxiety, and depression management, dietary modifications, weight management, and regular physical exercise) have demonstrated effectiveness in preventing and treating hypertension and should be considered to improve cardiovascular outcomes (48–53). Secondary treatment should be utilized, when appropriate, to reduce the risk of hypertension. Strategies to prevent and manage hypertension should address both clinical and structural barriers to effective care. The inclusion of community partners may help realize local strengths and resources and build the capacity necessary to improve outcomes.

### Limitations

While the current study featured a large sample size and sociodemographic representativeness of WWH in the US, the results may not be generalizable to men with HIV or WWH outside the WIHS. Furthermore, while WIHS participants reflect geographic diversity among 12 states, clustering of participants near study sites limits the generalizability of findings to more rural regions or areas outside the US. Additionally, the cross-sectional design does not allow for causal inferences and does not capture the cumulative impacts of adverse exposures to socioeconomic and psychosocial determinants over time. It also limits the ability to assess survival bias or account for resiliency factors that may influence long-term outcomes. Additional research is needed to identify sex-specific factors contributing to disparities in blood pressure and hypertension control among men and women with HIV and to better understand the cumulative effects of socioeconomic and psychosocial determinants on hypertension and its management over time. To reduce inequities, future research should aim to inform screening and treatment guidelines for people with HIV and evaluate the effectiveness and implementation of strategies designed to improve hypertension outcomes in this population.

## Conclusion

Exposure to adverse socioeconomic and psychosocial conditions may contribute to elevated blood pressure levels and an increased risk of hypertension among WWH and women at a heightened risk for HIV acquisition, increasing their risk of morbidity and mortality. Understanding how both area- and individual-level factors influence blood pressure and how women navigate and cope with stressors may inform interventions aimed at reducing disparities and improving health and quality of life outcomes. Although not demonstrated in this study, WWH may be particularly vulnerable to uncontrolled blood pressure levels, underscoring the need for future research to elucidate the pathways linking socioeconomic and psychosocial adversities to blood pressure outcomes. Understanding the long-term impact and pathways influencing outcomes is critical to the development of effective strategies to mitigate risk.

## Data Availability

The data analyzed in this study is subject to the following licenses/restrictions: MWCCS datasets are available upon request. Requests to access these datasets should be directed to mwccs@jhu.edu.

## References

[1] Alonso A Barnes AE Guest JL Shah A Shao IY Marconi V. HIV infection and incidence of cardiovascular diseases: an analysis of a large healthcare database. J Am Heart Assoc. (2019) 8:e012241. doi: 10.1161/jaha.119.012241, 31266386 PMC6662120

[2] Birabaharan M Strunk A Martin TCS. Burden of hypertension, diabetes, cardiovascular disease, and lung disease among women living with human immunodeficiency virus (HIV) in the United States. Clin Infect Dis. (2021) 73:169–70. doi: 10.1093/cid/ciaa1240, 32827257

[3] Collins LF Palella FJ Jr Mehta CC Holloway JN Stosor V Lake JE . Aging-related comorbidity burden among women and men with or at-risk for HIV in the US, 2008-2019. JAMA Netw Open. (2023) 6:–e2327584. doi: 10.1001/jamanetworkopen.2023.27584, 37548977 PMC10407688

[4] Sadinski LM Westreich D Edmonds A Breger TL Cole SR Ramirez C . Hypertension and one-year risk of all-cause mortality among women with treated HIV in the United States. AIDS. (2023) 37:679–88. doi: 10.1097/QAD.0000000000003461, 36728933 PMC9974900

[5] Siddiqui M Hannon L Wang Z Blair J Oparil S Heath SL . Hypertension and cardiovascular disease risk among individuals with versus without HIV. Hypertension (Dallas, Tex 1979). (2023) 80:852–60. doi: 10.1161/HYPERTENSIONAHA.122.19889, 36695187 PMC10023419

[6] Wise JM Jackson EA Kempf M-C Oates GR Wang Z Overton ET. Sex differences in incident atherosclerotic cardiovascular disease events among women and men with HIV. AIDS. (2023) 37:1661–9. doi: 10.1097/QAD.0000000000003592, 37195280

[7] Fahme SA Bloomfield GS Peck R. Hypertension in HIV-infected adults. Hypertension. (2018) 72:44–55. doi: 10.1161/HYPERTENSIONAHA.118.10893, 29776989 PMC6002926

[8] Stone L Looby SE Zanni MV. Cardiovascular disease risk among women living with HIV in North America and Europe. Curr Opin HIV AIDS. (2017) 12:585–93. doi: 10.1097/coh.0000000000000413, 28832367 PMC6002961

[9] Fioranelli M Bottaccioli AG Bottaccioli F Bianchi M Rovesti M Roccia MG. Stress and inflammation in coronary artery disease: a review Psychoneuroendocrineimmunology-based. Front Immunol. (2018) 9:2031–1. doi: 10.3389/fimmu.2018.02031, 30237802 PMC6135895

[10] Inoue N. Stress and atherosclerotic cardiovascular disease. J Atheroscler Thromb. (2014) 21:391–401. doi: 10.5551/jat.21709 24561512

[11] Pellowski JA Pellowski JA Huedo-Medina TB Huedo-Medina TB Kalichman SC Kalichman SC. Food insecurity, substance use, and sexual transmission risk behavior among people living with HIV: a daily level analysis. Arch Sex Behav. (2018) 47:1899–907. doi: 10.1007/s10508-017-0942-4, 28429158 PMC5650554

[12] Phelan JC Link BG Tehranifar P. Social conditions as fundamental causes of health inequalities: theory, evidence, and policy implications. J Health Soc Behav. (2010) 51:S28–40. doi: 10.1177/0022146510383498, 20943581

[13] Steptoe A Kivimäki M. Stress and cardiovascular disease. Nat Rev Cardiol. (2012) 9:360–70. doi: 10.1038/nrcardio.2012.45, 22473079

[14] Turner RJ. Understanding health disparities: the relevance of the stress process model. Soc Ment Health. (2013) 3:170–86. doi: 10.1177/2156869313488121

[15] Yao B-C Meng L-B Hao M-L Zhang Y-M Gong T Guo Z-G. Chronic stress: a critical risk factor for atherosclerosis. J Int Med Res. (2019) 47:1429–40. doi: 10.1177/0300060519826820, 30799666 PMC6460614

[16] Adimora AA Ramirez C Benning L . Cohort profile: the women's interagency HIV study (WIHS). Int J Epidemiol. (2018) 47:393–394i. doi: 10.1093/ije/dyy021 29688497 PMC5913596

[17] Vidal-Petiot E. Thresholds for hypertension definition, treatment initiation, and treatment targets: recent guidelines at a glance. Circulation 2022;146:805–807. doi:doi: 10.1161/CIRCULATIONAHA.121.055177 36095063

[18] Jones DW Ferdinand KC Taler SJ Johnson HM Shimbo D Abdalla M . 2025 AHA/ACC/AANP/AAPA/ABC/ACCP/ACPM/AGS/AMA/ASPC/NMA/PCNA/SGIM guideline for the prevention, detection, evaluation, and management of high blood pressure in adults. JACC. (2025) 86:1567–678. doi: 10.1016/j.jacc.2025.05.007, 40815242

[19] Cutter SL. The origin and diffusion of the social vulnerability index (SoVI). Int J Disaster Risk Reduct. (2024) 109:104576. doi: 10.1016/j.ijdrr.2024.104576

[20] Brown MJ Gao C Kaur A Qiao S Li X. Social support, internalized HIV stigma, resilience and depression among people living with HIV: a moderated mediation analysis. AIDS Behav. (2023) 27:1106–15. doi: 10.1007/s10461-022-03847-7, 36094638 PMC10115436

[21] Chandran A Benning L Musci RJ Wilson TE Milam J Adedimeji A . The longitudinal association between social support on HIV medication adherence and healthcare utilization in the women's interagency HIV study. AIDS Behav. (2019) 23:2014–24. doi: 10.1007/s10461-018-2308-x, 30311104 PMC7331802

[22] Bunn JY Solomon SE Miller C Forehand R. Measurement of stigma in people with HIV: a reexamination of the HIV stigma scale. AIDS Educ Prev. (2007) 19:198–208. doi: 10.1521/aeap.2007.19.3.198, 17563274

[23] Arnett DK Blumenthal RS Albert MA Buroker AB Goldberger ZD Hahn EJ . 2019 ACC/AHA guideline on the primary prevention of cardiovascular disease: a report of the American College of Cardiology/American Heart Association task force on clinical practice guidelines. Circulation. (2019) 140:e596–646. doi: 10.1161/cir.0000000000000678 30879355 PMC7734661

[24] Feinstein MJ Hsue PY Benjamin LA Bloomfield GS Currier JS Freiberg MS . Characteristics, prevention, and management of cardiovascular disease in people living with HIV: a scientific statement from the American Heart Association. Circulation. (2019) 140:e98–e124. doi: 10.1161/CIR.0000000000000695, 31154814 PMC7993364

[25] Thompson FE Midthune D Subar AF McNeel T Berrigan D Kipnis V. Dietary intake estimates in the National Health Interview Survey, 2000: methodology, results, and interpretation. J Am Diet Assoc. (2005) 105:352–63. doi: 10.1016/j.jada.2004.12.032, 15746822

[26] Rubin LH Gustafson DR Warrior L Sheira L Fitzgerald KC Dastgheyb R . Dietary intake is associated with neuropsychological impairment in women with HIV. Am J Clin Nutr. (2021) 114:378–89. doi: 10.1093/ajcn/nqab038, 33829235 PMC8246600

[27] Sidney S Jacobs DR Jr Haskell WL Armstrong MA Dimicco A Oberman A . Comparison of two methods of assessing physical activity in the coronary artery risk development in young adults (CARDIA) study. Am J Epidemiol. (1991) 133:1231–45. doi: 10.1093/oxfordjournals.aje.a115835, 2063831

[28] Tien PC Benson C Zolopa AR Sidney S Osmond D Grunfeld C. The study of fat redistribution and metabolic change in HIV infection (FRAM): methods, design, and sample characteristics. Am J Epidemiol. (2006) 163:860–9. doi: 10.1093/aje/kwj111, 16524955 PMC3170407

[29] Control CfD. 8 or more drinks per week is a heavy drinker for females. (2025). Accessed 7/11/14, Available online at: www.cdc.gov/alcohol/fact-sheets/alcohol-use.html

[30] Radloff L. The CES-D scale: a self-report depression scale for research in the general population. Appl Psychol Meas. (1977) 1:384–401. doi: 10.1177/014662167700100306

[31] Cohen S Kamarck T Mermelstein R. A global measure of perceived stress. J Health Soc Behav. (1983) 24:385–96. doi: 10.2307/2136404, 6668417

[32] Weathers F Litz B Herman D Huska JA Keane T. The PTSD checklist (PCL): reliability, validity, and diagnostic utility. Paper Presented at the Annual Convention of the International Society for Traumatic Stress Studies. 1993;

[33] SAS Institute; 2013. SAS 9.4 Software.

[34] R Core Team. R: a language and environment for statistical computing. R Foundation for Statistical Computing. Vienna. (2023). Available online at: https://www.R-project.org/

[35] Weng X Kompaniyets L Buchacz K Thompson-Paul AM Woodruff RC Hoover KW . Hypertension prevalence and control among people with and without HIV — United States, 2022. Am J Hypertens. (2024) 37:661–6. doi: 10.1093/ajh/hpae048 38668635

[36] Blair J Kempf M-C Dionne JA Causey-Pruitt Z Wise JM Jackson EA . Awareness, treatment, and control of hypertension among women at risk or living with HIV in the US south. AIDS. (2024) 38:1703–13. doi: 10.1097/QAD.0000000000003960, 38905486 PMC11293969

[37] Lines LM Urato M Halpern MT Subramanian S. (2014). RTI press research report series. Insurance coverage and preventive care among adults. RTI press © 2014 Research Triangle Institute. All rights reserved. 2014. doi: 10.3768/rtipress.2014.rr.0021.1404 30354043

[38] Muntner P Hardy ST Fine LJ Jaeger BC Wozniak G Levitan EB . Trends in blood pressure control among US adults with hypertension, 1999-2000 to 2017-2018. JAMA. (2020) 324:1190–200. doi: 10.1001/jama.2020.14545, 32902588 PMC7489367

[39] Olaiya O Weiser J Zhou W Patel P Bradley H. Hypertension among persons living with HIV in medical care in the United States-medical monitoring project, 2013-2014. Open Forum Infect Dis. (2018) 5:ofy028. doi: 10.1093/ofid/ofy028, 29516021 PMC5833317

[40] Ruffieux Y Joska JA Lang R Zheng C Folb N Kirk GD . Life-years lost associated with mental disorders in people with HIV: a cohort study in South Africa, Canada and the United States. J Int AIDS Soc. (2025) 28:e70023. doi: 10.1002/jia2.70023, 40826829 PMC12361348

[41] Gill A Clum G Molina P Welsh D Ferguson T Theall KP. Life course stressors, latent coping strategies, alcohol use, and adherence among people with HIV. AIDS Behav. (2024) 29:589–99. doi: 10.1007/s10461-024-04541-6, 39546146 PMC11814058

[42] Brownley JR Fallot RD Wolfson Berley R Himelhoch SS. Trauma history in African-American women living with HIV: effects on psychiatric symptom severity and religious coping. AIDS Care. (2015) 27:964–71. doi: 10.1080/09540121.2015.1017441, 25742054

[43] Waldron EM Burnett-Zeigler I Wee V Ng YW Koenig LJ Pederson AB . Mental health in women living with HIV: the unique and unmet needs. J Int Assoc Provid AIDS Care. (2021) 20:2325958220985665. doi: 10.1177/2325958220985665, 33472517 PMC7829520

[44] Denison JA Willis K DeLong SM Sievwright KM Agwu AL Arrington-Sanders R . Advancing adolescent and young adult HIV prevention and care and treatment through use of multi-level theories and frameworks: a scoping review and adapted HIV ecological framework. AIDS Behav. (2024) 28:1694–707. doi: 10.1007/s10461-023-04255-1, 38351279 PMC11069483

[45] Whelton PK Carey RM Aronow WS Casey de Jr Collins KJ Dennison Himmelfarb C . 2017 ACC/AHA/AAPA/ABC/ACPM/AGS/APhA/ASH/ASPC/NMA/PCNA guideline for the prevention, detection, evaluation, and Management of High Blood Pressure in adults: a report of the American College of Cardiology/American Heart Association task force on clinical practice guidelines. J Am Coll Cardiol. (2018) 71:e127–248. doi: 10.1016/j.jacc.2017.11.006, 29146535

[46] Armah KA Chang CC Baker JV Ramachandran VS Budoff MJ Crane HM . Prehypertension, hypertension, and the risk of acute myocardial infarction in HIV-infected and -uninfected veterans. Clin Infect Dis. (2014) 58:121–9. doi: 10.1093/cid/cit652, 24065316 PMC3864500

[47] Nüesch R Wang Q Elzi L Bernasconi E Weber R Cavassini M . Risk of cardiovascular events and blood pressure control in hypertensive HIV-infected patients: Swiss HIV cohort study (SHCS). JAIDS J Acquir Immune Defic Syndr. (2013) 62:396–404. doi: 10.1097/QAI.0b013e3182847cd0, 23288033

[48] Blumenthal JA Hinderliter AL Smith PJ Mabe S Watkins LL Craighead L . Effects of lifestyle modification on patients with resistant hypertension: results of the TRIUMPH randomized clinical trial. Circulation. (2021) 144:1212–26. doi: 10.1161/CIRCULATIONAHA.121.055329, 34565172 PMC8511053

[49] Filippou CD Tsioufis CP Thomopoulos CG Mihas CC Dimitriadis KS Sotiropoulou LI . Dietary approaches to stop hypertension (DASH) diet and blood pressure reduction in adults with and without hypertension: a systematic review and meta-analysis of randomized controlled trials. Adv Nutr. (2020) 11:1150–60. doi: 10.1093/advances/nmaa041, 32330233 PMC7490167

[50] Kalinowski J Kaur K Newsome-Garcia V Langford A Kalejaiye A Vieira D . Stress interventions and hypertension in black women. Womens Health. (2021) 17:17455065211009751–1. doi: 10.1177/17455065211009751, 34254559 PMC8280834

[51] Ozemek C Tiwari S Sabbahi A Carbone S Lavie CJ. Impact of therapeutic lifestyle changes in resistant hypertension. Prog Cardiovasc Dis. (2020) 63:4–9. doi: 10.1016/j.pcad.2019.11.012, 31756356 PMC7257910

[52] Pescatello LS Buchner DM Jakicic JM Powell KE Kraus WE Bloodgood B . Physical activity to prevent and treat hypertension: a systematic review. Med Sci Sports Exerc. (2019) 51:1314–23. doi: 10.1249/mss.0000000000001943, 31095088

[53] Valenzuela PL Carrera-Bastos P Gálvez BG Ruiz-Hurtado G Ordovas JM Ruilope LM . Lifestyle interventions for the prevention and treatment of hypertension. Nat Rev Cardiol. (2021) 18:251–75. doi: 10.1038/s41569-020-00437-9, 33037326

[54] Wise J. (2025). Socioecological Model of Stress and Cardiovascular Disease Risk. Created in BioRender. https://BioRender.com/m74jtsw

